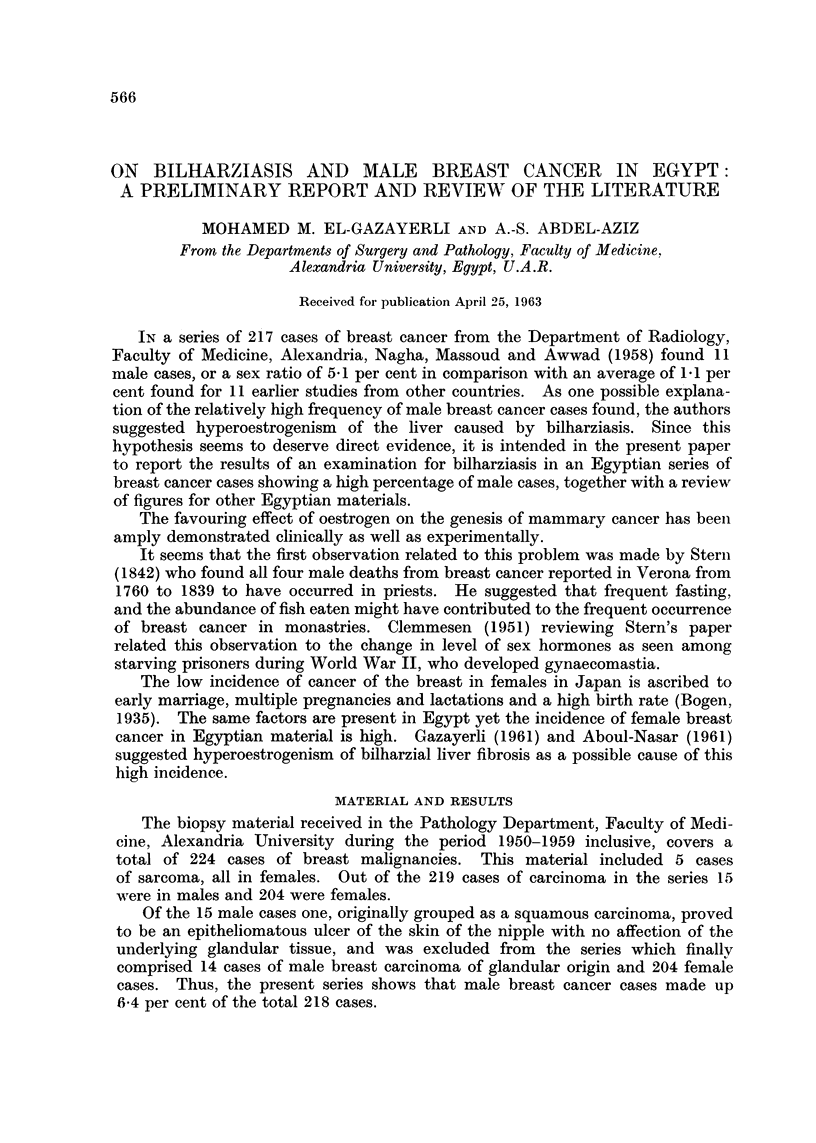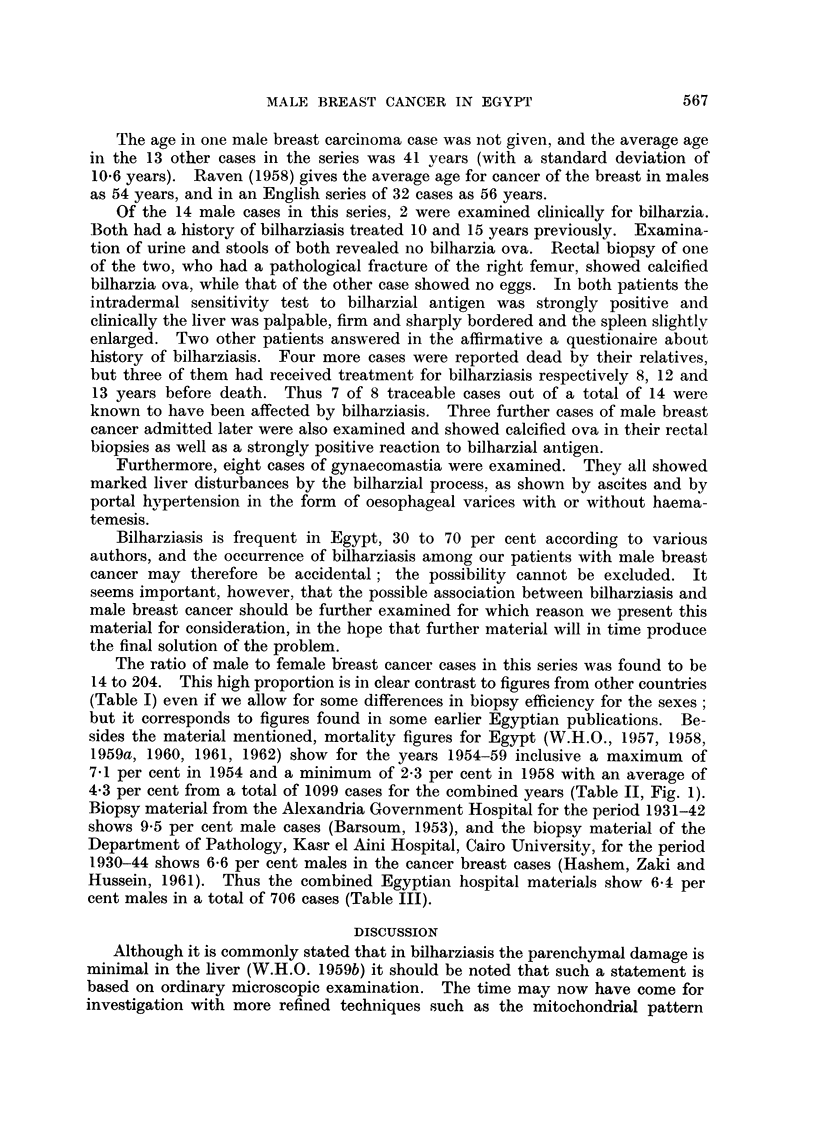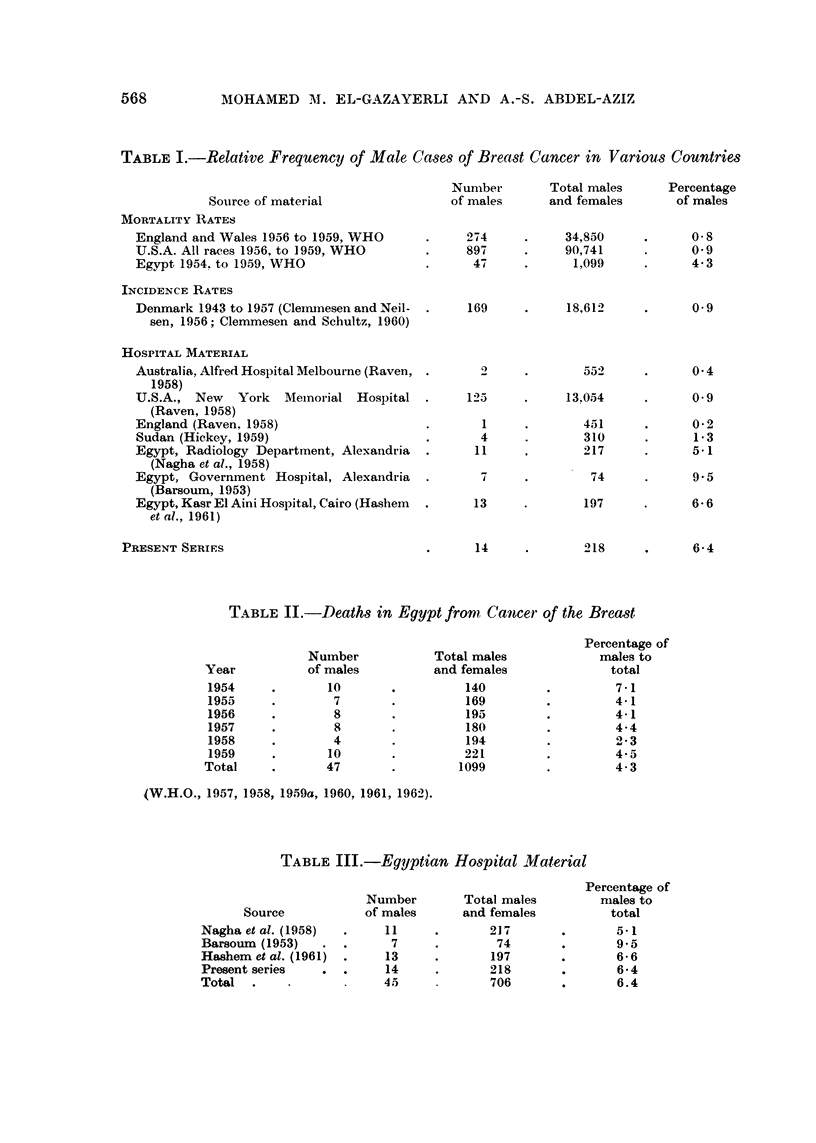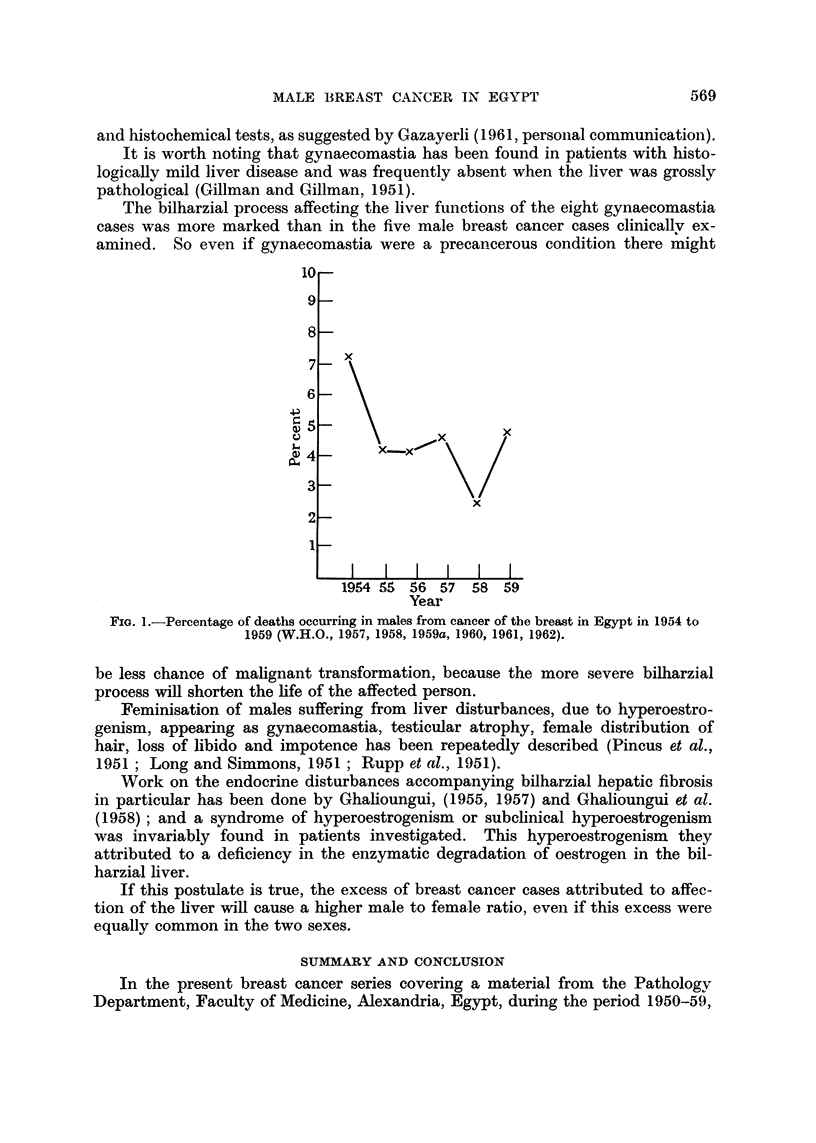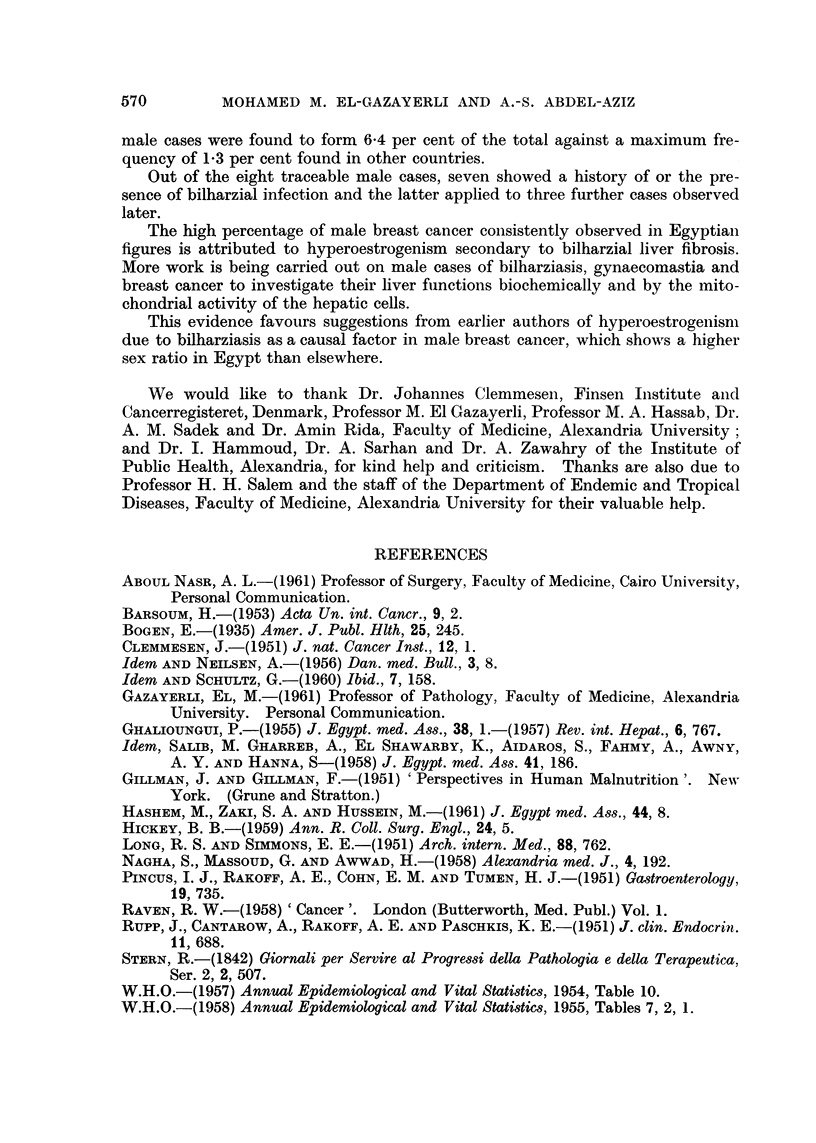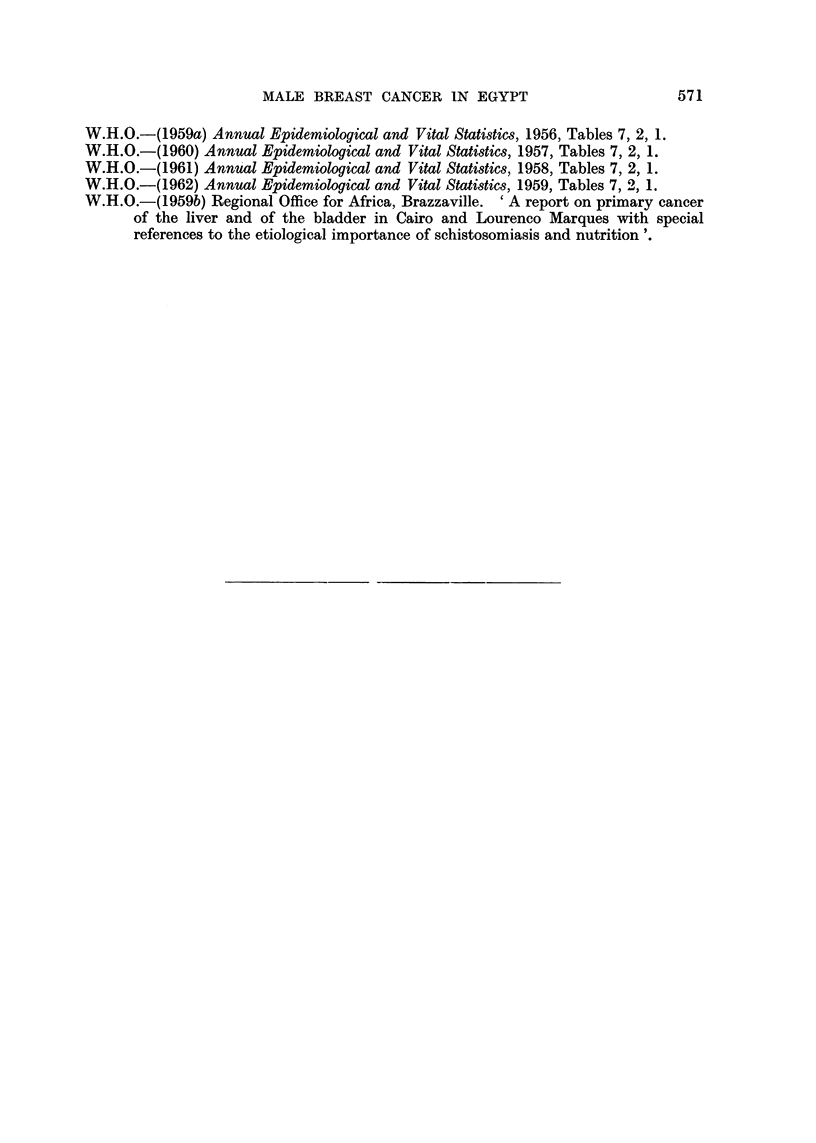# On Bilharziasis and Male Breast Cancer in Egypt: A Preliminary Report and Review of the Literature

**DOI:** 10.1038/bjc.1963.75

**Published:** 1963-12

**Authors:** Mohamed M. El-Gazayerli, A.-S. Abdel-Aziz


### ON BILHARZIASIS AND MALE BREAST CANCER IN EGYPT: A PRELIMINARY REPORT AND REVIEW OF THE LITERATURE

MOHAMED M. EL-GAZAYERLI AND A.-S. ABDEL-AZIZ

*From the Departments of Surgery and Pathology, Faculty of Medicine, Alexandria University, Egypt, U.A.R.*

Received for publication April 25, 1963

IN a series of 217 cases of breast cancer from the Department of Radiology, Faculty of Medicine, Alexandria, Nagha, Massoud and Awwad (1958) found 11 male cases, or a sex ratio of 5·1 per cent in comparison with an average of 1·1 per cent found for 11 earlier studies from other countries. As one possible explanation of the relatively high frequency of male breast cancer cases found, the authors suggested hyperoestrogenism of the liver caused by bilharziasis. Since this hypothesis seems to deserve direct evidence, it is intended in the present paper to report the results of an examination for bilharziasis in an Egyptian series of breast cancer cases showing a high percentage of male cases, together with a review of figures for other Egyptian materials.

The favouring effect of oestrogen on the genesis of mammary cancer has been amply demonstrated clinically as well as experimentally.

It seems that the first observation related to this problem was made by Stern (1842) who found all four male deaths from breast cancer reported in Verona from 1760 to 1839 to have occurred in priests. He suggested that frequent fasting, and the abundance of fish eaten might have contributed to the frequent occurrence of breast cancer in monastries. Clemmesen (1951) reviewing Stern's paper related this observation to the change in level of sex hormones as seen among starving prisoners during World War II, who developed gynaecomastia.

The low incidence of cancer of the breast in females in Japan is ascribed to early marriage, multiple pregnancies and lactations and a high birth rate (Bogen, 1935). The same factors are present in Egypt yet the incidence of female breast cancer in Egyptian material is high. Gazayerli (1961) and Aboul-Nasar (1961) suggested hyperoestrogenism of bilharzial liver fibrosis as a possible cause of this high incidence.

### MATERIAL AND RESULTS

The biopsy material received in the Pathology Department, Faculty of Medicine, Alexandria University during the period 1950–1959 inclusive, covers a total of 224 cases of breast malignancies. This material included 5 cases of sarcoma, all in females. Out of the 219 cases of carcinoma in the series 15 were in males and 204 were females.

Of the 15 male cases one, originally grouped as a squamous carcinoma, proved to be an epitheliomatous ulcer of the skin of the nipple with no affection of the underlying glandular tissue, and was excluded from the series which finally comprised 14 cases of male breast carcinoma of glandular origin and 204 female cases. Thus, the present series shows that male breast cancer cases made up 6·4 per cent of the total 218 cases.

The age in one male breast carcinoma case was not given, and the average age in the 13 other cases in the series was 41 years (with a standard deviation of 10·6 years). Raven (1958) gives the average age for cancer of the breast in males as 54 years, and in an English series of 32 cases as 56 years.

Of the 14 male cases in this series, 2 were examined clinically for bilharzia. Both had a history of bilharziasis treated 10 and 15 years previously. Examination of urine and stools of both revealed no bilharzia ova. Rectal biopsy of one of the two, who had a pathological fracture of the right femur, showed calcified bilharzia ova, while that of the other case showed no eggs. In both patients the intradermal sensitivity test to bilharzial antigen was strongly positive and clinically the liver was palpable, firm and sharply bordered and the spleen slightly enlarged. Two other patients answered in the affirmative a questionaire about history of bilharziasis. Four more cases were reported dead by their relatives, but three of them had received treatment for bilharziasis respectively 8, 12 and 13 years before death. Thus 7 of 8 traceable cases out of a total of 14 were known to have been affected by bilharziasis. Three further cases of male breast cancer admitted later were also examined and showed calcified ova in their rectal biopsies as well as a strongly positive reaction to bilharzial antigen.

Furthermore, eight cases of gynaecomastia were examined. They all showed marked liver disturbances by the bilharzial process, as shown by ascites and by portal hypertension in the form of oesophageal varices with or without haematemesis.

Bilharziasis is frequent in Egypt, 30 to 70 per cent according to various authors, and the occurrence of bilharziasis among our patients with male breast cancer may therefore be accidental ; the possibility cannot be excluded. It seems important, however, that the possible association between bilharziasis and male breast cancer should be further examined for which reason we present this material for consideration, in the hope that further material will in time produce the final solution of the problem.

The ratio of male to female breast cancer cases in this series was found to be 14 to 204. This high proportion is in clear contrast to figures from other countries (Table I) even if we allow for some differences in biopsy efficiency for the sexes ; but it corresponds to figures found in some earlier Egyptian publications. Besides the material mentioned, mortality figures for Egypt (W.H.O., 1957, 1958, 1959*a*, 1960, 1961, 1962) show for the years 1954–59 inclusive a maximum of 7·1 per cent in 1954 and a minimum of 2·3 per cent in 1958 with an average of 4·3 per cent from a total of 1099 cases for the combined years (Table II, Fig. 1). Biopsy material from the Alexandria Government Hospital for the period 1931–42 shows 9·5 per cent male cases (Barsoum, 1953), and the biopsy material of the Department of Pathology, Kasr el Aini Hospital, Cairo University, for the period 1930–44 shows 6·6 per cent males in the cancer breast cases (Hashem, Zaki and Hussein, 1961). Thus the combined Egyptian hospital materials show 6·4 per cent males in a total of 706 cases (Table III).

### DISCUSSION

Although it is commonly stated that in bilharziasis the parenchymal damage is minimal in the liver (W.H.O. 1959*b*) it should be noted that such a statement is based on ordinary microscopic examination. The time may now have come for investigation with more refined techniques such as the mitochondrial pattern

TABLE I.—*Relative Frequency of Male Cases of Breast Cancer in Various Countries*

| Source of material | Number of males | Total males and females | Percentage of males |
|---|---|---|---|
| MORTALITY RATES | | | |
| England and Wales 1956 to 1959, WHO | 274 | 34,850 | 0·8 |
| U.S.A. All races 1956, to 1959, WHO | 897 | 90,741 | 0·9 |
| Egypt 1954, to 1959, WHO | 47 | 1,099 | 4·3 |
| INCIDENCE RATES | | | |
| Denmark 1943 to 1957 (Clemmesen and Neilsen, 1956; Clemmesen and Schultz, 1960) | 169 | 18,612 | 0·9 |
| HOSPITAL MATERIAL | | | |
| Australia, Alfred Hospital Melbourne (Raven, 1958) | 2 | 552 | 0·4 |
| U.S.A., New York Memorial Hospital (Raven, 1958) | 125 | 13,054 | 0·9 |
| England (Raven, 1958) | 1 | 451 | 0·2 |
| Sudan (Hickey, 1959) | 4 | 310 | 1·3 |
| Egypt, Radiology Department, Alexandria (Nagha *et al.*, 1958) | 11 | 217 | 5·1 |
| Egypt, Government Hospital, Alexandria (Barsoum, 1953) | 7 | 74 | 9·5 |
| Egypt, Kasr El Aini Hospital, Cairo (Hashem *et al.*, 1961) | 13 | 197 | 6·6 |
| PRESENT SERIES | 14 | 218 | 6·4 |

TABLE II.—*Deaths in Egypt from Cancer of the Breast*

| Year | Number of males | Total males and females | Percentage of males to total |
|---|---|---|---|
| 1954 | 10 | 140 | 7·1 |
| 1955 | 7 | 169 | 4·1 |
| 1956 | 8 | 195 | 4·1 |
| 1957 | 8 | 180 | 4·4 |
| 1958 | 4 | 194 | 2·3 |
| 1959 | 10 | 221 | 4·5 |
| Total | 47 | 1099 | 4·3 |

(W.H.O., 1957, 1958, 1959*a*, 1960, 1961, 1962).

TABLE III.—*Egyptian Hospital Material*

| Source | Number of males | Total males and females | Percentage of males to total |
|---|---|---|---|
| Nagha *et al.* (1958) | 11 | 217 | 5·1 |
| Barsoum (1953) | 7 | 74 | 9·5 |
| Hashem *et al.* (1961) | 13 | 197 | 6·6 |
| Present series | 14 | 218 | 6·4 |
| Total | 45 | 706 | 6.4 |

and histochemical tests, as suggested by Gazayerli (1961, personal communication).

It is worth noting that gynaecomastia has been found in patients with histologically mild liver disease and was frequently absent when the liver was grossly pathological (Gillman and Gillman, 1951).

The bilharzial process affecting the liver functions of the eight gynaecomastia cases was more marked than in the five male breast cancer cases clinically examined. So even if gynaecomastia were a precancerous condition there might

FIG. 1.—Percentage of deaths occurring in males from cancer of the breast in Egypt in 1954 to 1959 (W.H.O., 1957, 1958, 1959*a*, 1960, 1961, 1962).

be less chance of malignant transformation, because the more severe bilharzial process will shorten the life of the affected person.

Feminisation of males suffering from liver disturbances, due to hyperoestrogenism, appearing as gynaecomastia, testicular atrophy, female distribution of hair, loss of libido and impotence has been repeatedly described (Pincus *et al.*, 1951 ; Long and Simmons, 1951 ; Rupp *et al.*, 1951).

Work on the endocrine disturbances accompanying bilharzial hepatic fibrosis in particular has been done by Ghalioungui, (1955, 1957) and Ghalioungui *et al.* (1958) ; and a syndrome of hyperoestrogenism or subclinical hyperoestrogenism was invariably found in patients investigated. This hyperoestrogenism they attributed to a deficiency in the enzymatic degradation of oestrogen in the bilharzial liver.

If this postulate is true, the excess of breast cancer cases attributed to affection of the liver will cause a higher male to female ratio, even if this excess were equally common in the two sexes.

### SUMMARY AND CONCLUSION

In the present breast cancer series covering a material from the Pathology Department, Faculty of Medicine, Alexandria, Egypt, during the period 1950–59, male cases were found to form 6·4 per cent of the total against a maximum frequency of 1·3 per cent found in other countries.

Out of the eight traceable male cases, seven showed a history of or the presence of bilharzial infection and the latter applied to three further cases observed later.

The high percentage of male breast cancer consistently observed in Egyptian figures is attributed to hyperoestrogenism secondary to bilharzial liver fibrosis. More work is being carried out on male cases of bilharziasis, gynaecomastia and breast cancer to investigate their liver functions biochemically and by the mitochondrial activity of the hepatic cells.

This evidence favours suggestions from earlier authors of hyperoestrogenism due to bilharziasis as a causal factor in male breast cancer, which shows a higher sex ratio in Egypt than elsewhere.

We would like to thank Dr. Johannes Clemmesen, Finsen Institute and Cancerregisteret, Denmark, Professor M. El Gazayerli, Professor M. A. Hassab, Dr. A. M. Sadek and Dr. Amin Rida, Faculty of Medicine, Alexandria University; and Dr. I. Hammoud, Dr. A. Sarhan and Dr. A. Zawahry of the Institute of Public Health, Alexandria, for kind help and criticism. Thanks are also due to Professor H. H. Salem and the staff of the Department of Endemic and Tropical Diseases, Faculty of Medicine, Alexandria University for their valuable help.

### REFERENCES

Aboul Nasr, A. L.—(1961) Professor of Surgery, Faculty of Medicine, Cairo University, Personal Communication.

Barsoum, H.—(1953) *Acta Un. int. Cancr.*, **9**, 2.

Bogen, E.—(1935) *Amer. J. Publ. Hlth*, **25**, 245.

Clemmesen, J.—(1951) *J. nat. Cancer Inst.*, **12**, 1.

*Idem* and Neilsen, A.—(1956) *Dan. med. Bull.*, **3**, 8.

*Idem* and Schultz, G.—(1960) *Ibid.*, **7**, 158.

Gazayerli, El, M.—(1961) Professor of Pathology, Faculty of Medicine, Alexandria University. Personal Communication.

Ghalioungui, P.—(1955) *J. Egypt. med. Ass.*, **38**, 1.—(1957) *Rev. int. Hepat.*, **6**, 767.

*Idem*, Salib, M. Gharreb, A., El Shawarby, K., Aidaros, S., Fahmy, A., Awny, A. Y. and Hanna, S—(1958) *J. Egypt. med. Ass.* **41**, 186.

Gillman, J. and Gillman, F.—(1951) ' Perspectives in Human Malnutrition '. New York. (Grune and Stratton.)

Hashem, M., Zaki, S. A. and Hussein, M.—(1961) *J. Egypt med. Ass.*, **44**, 8.

Hickey, B. B.—(1959) *Ann. R. Coll. Surg. Engl.*, **24**, 5.

Long, R. S. and Simmons, E. E.—(1951) *Arch. intern. Med.*, **88**, 762.

Nagha, S., Massoud, G. and Awwad, H.—(1958) *Alexandria med. J.*, **4**, 192.

Pincus, I. J., Rakoff, A. E., Cohn, E. M. and Tumen, H. J.—(1951) *Gastroenterology*, **19**, 735.

Raven, R. W.—(1958) ' Cancer '. London (Butterworth, Med. Publ.) Vol. 1.

Rupp, J., Cantarow, A., Rakoff, A. E. and Paschkis, K. E.—(1951) *J. clin. Endocrin.* **11**, 688.

Stern, R.—(1842) *Giornali per Servire al Progressi della Pathologia e della Terapeutica*, Ser. 2, **2**, 507.

W.H.O.—(1957) *Annual Epidemiological and Vital Statistics*, 1954, Table 10.

W.H.O.—(1958) *Annual Epidemiological and Vital Statistics*, 1955, Tables 7, 2, 1.

W.H.O.—(1959*a*) *Annual Epidemiological and Vital Statistics*, 1956, Tables 7, 2, 1.
W.H.O.—(1960) *Annual Epidemiological and Vital Statistics*, 1957, Tables 7, 2, 1.
W.H.O.—(1961) *Annual Epidemiological and Vital Statistics*, 1958, Tables 7, 2, 1.
W.H.O.—(1962) *Annual Epidemiological and Vital Statistics*, 1959, Tables 7, 2, 1.
W.H.O.—(1959*b*) Regional Office for Africa, Brazzaville. ' A report on primary cancer of the liver and of the bladder in Cairo and Lourenco Marques with special references to the etiological importance of schistosomiasis and nutrition '.